# Expression of gamma-aminobutyric acid receptors on neoplastic growth and prediction of prognosis in non-small cell lung cancer

**DOI:** 10.1186/1479-5876-11-102

**Published:** 2013-04-24

**Authors:** Xiaoxue Zhang, Rong Zhang, Yuanjie Zheng, Jianfei Shen, Dakai Xiao, Jin Li, Xiaoshun Shi, Liyan Huang, Hailing Tang, Jun Liu, Jianxing He, Haibo Zhang

**Affiliations:** 1 Guangdong Cardiovascular Institute, Southern Medical University, Guangzhou, Guangdong Province, 510080, China; 2 Guangzhou Research Institute of Respiratory Disease & China State Key Laboratory of Respiratory Disease, The First Affiliated Hospital of Guangzhou Medical College, Guangzhou, Guangdong Province, 510120, China; 3 Departments of Anesthesia, Medicine and Physiology, University of Toronto, Toronto, Ontario, M5B 1W8, Canada

**Keywords:** Gamma aminobutyric acid receptor, Survival, Biomarker, Prognosis

## Abstract

**Background:**

Gamma-aminobutyric acid (GABA) is the main inhibitory neurotransmitter in the adult mammalian brain, but exerts physiologic effects other than that on neurotransmitter in non-neuronal peripheral tissues and organs. GABA may affect cancer growth through activation GABA receptors. We investigated the gene expression of GABA receptors in tissue of non-small cell lung cancers (NSCLC) and non-cancerous tissues, and found that the gene expression of GABA receptor phenotypes was correlated with tumorigenesis and clinical prognosis.

**Methods:**

Sixty-one snap-frozen human samples of NSCLC tissues and paired non-cancerous tissues (5cm away from tumor) were analyzed. Gene expression of GABA receptors was detected by Real-time quantitative PCR (RT-qPCR). Survival times in relation to the expression of GABA receptor phenotypes were analyzed. Human NSCLC cell lines H1299, A549, H520, H460 and human bronchial epithelial cell line BEAS-2B were used to determine the phenotypes of GABA inhibitory effects on cancer cell growth. The effects of exogenous administration of GABA on H1299 cell growth were examined.

**Results:**

The gene expressions were significantly higher in NSCLC tissues than in the paired non-cancerous tissues for GABA_A_ receptor subunit α3 (GABR_A3_, *P* = 0.030); for GABA_A_ receptor subunit epsilon (GABR_E_, *P* = 0.036); and GABA_B_ receptor subunit 2 (GABBR_2_, *P* = 0.005). Kaplan-Meier curves showed that patients with high expression of GABBR_2_ gene and low expression of GABR_A3_ gene had a better prognosis (P < 0.05). The administration of GABA resulted in suppressed proliferation of NSCLC cell lines in a dose- and time-dependent manner. The use of the GABA receptor antagonist CGP35348 could reverse the inhibitory effect.

**Conclusions:**

The pattern of GABA receptor gene phenotype expression may be involved in the regulation of tumorigenesis. A high expression of GABBR_2_ with a low expression of GABR_A3_ may predict a better outcome. The treatment with GABA attenuates cancer cell growth in vitro. The expression of GABA receptor may be not only promising genetic therapeutic targets but may also serve as valuable prognostic markers for NSCLC.

## Background

Cancer is a major global public health problem. One in 4 deaths in the United States is due to cancer [1]. Lung cancer is comprising 17% of the total new cancer cases and 23% of the total cancer deaths [2]. Non-small cell lung cancer (NSCLC) accounts for about 80% of all lung cancer cases where adenocarcinoma is dominantly presented [3]. Conventional treatment of NSCLC has improved survival, but the 5-year survival rate is approximately 16% over the past 30 years [1]. Novel and effective methods are urgently required for lung cancer therapy.

Gamma-aminobutyric acid (GABA) is the main inhibitory neurotransmitter in the adult mammalian brain via activation of specific GABA receptors highly expressed in the central nervous system (CNS) [4,5]. GABA receptors are composed of GABA_A_ and GABA_B_ receptors. GABA_A_ receptors are ligand-gated chloride channels composed of five subunits. These subunits are encoded by 19 different genes that have been grouped into eight subclasses based on sequence homology (α1–6, β1–3, γ1–3, δ, ϵ, θ, π, ρ1–3). GABA_B_ receptors are heterodimeric G-protein-coupled receptors (GPCRs) composed of GABBR_1_ and GABBR_2_ subunits which are both required for normal receptor functioning [6].

It has recently revealed that GABA and its receptors also exist in non-neuronal peripheral tissues and organs, indicating that GABA exerts physiologic effects other than the inhibitory neurotransmitter property. In fact, GABA has been shown to be involved in the development of many tissues and organs, including the peripheral nervous system [7], the development of the palate [8], lung [9], pancreas [10], digestive tract [11], liver [12], chondrocytes [13], testicular cells [14] and even stem cells [15].

Given that GABA participates in the proliferation of various normal cell types and tissues, it is intriguing to consider the potential function of GABA in cancer cells. Recent studies gave the evidences that GABA and its receptors seemed to play critically regulative effects on many kinds of cancers [12,16-31]. In most cases, the levels of GABA receptors accompanying other growth signaling components had significant changes in cancer cells. This raised the possibility that manipulating GABA receptor activity might inhibit tumor growth [25].

In this study, we tested the hypothesis that GABA receptor profiles modulate cancer survival. We thus investigated the gene expression of GABA receptor phenotypes in NSCLC tissues and paired non-cancerous tissues obtained from surgical patients to correlate the GABA receptor gene profiles with clinical outcome. To examine the specific effects of GABA receptor on lung cancer cell growth, we investigated the GABA receptor profiles in cancer cell lines and in normal human epithelial cell line in the presence and absence of exogenous administration of GABA.

## Methods

### Cancer cell lines

Human NSCLC cell lines H1299(adenocarcinoma), A549(adenocarcinoma), H520(squamous cell carcinoma), H460(large cell carcinoma) and normal human bronchial epithelial cell line BEAS-2B (ATCC, Rockville, Maryland) were cultured at 37°C with 5% CO_2_ in DMEM (Gibco, Beijing, China) supplemented with 10% FBS (Gibco, Beijing, China) without antibiotics.

### MTT assays for cell proliferation

To measure the proliferation of cells, the colorimetric 3-(4, 5-dimethyle thiazol-2-yl)-2, 5-diphenyl tetrazolium bromide (MTT) assays (Sigma) were used in vitro. In the MTT assays, cells were seeded into 96-well plates (2 × 10^3^ cells per well), grown overnight, washed in PBS, and incubated with GABA (Sigma-Aldrich) at 0.1 μmol/L to 500 μmol/L in the presence or absence of 100 μmol/L picrotoxin (PTX, GABA_A_ receptor antagonist) or CGP35348 (CGP, GABA_B_ receptor antagonist), respectively. MTT was then added (10ug/well) for 4h. Formazan products were solubilized with DMSO, and the optical density was measured at 490 nm.

### Tissues and subjects

Sixty-one samples of NSCLC tissues and paired non-cancerous tissues (5cm away from tumor) were collected from the Thoracic Cardio Surgery Department of the First Affiliated Hospital of Guangzhou Medical College under full ethical clearance by the Guangzhou Medical College Ethics Committee for experimentation on human subjects. Informed written consent was obtained from the participants. All subjects were Chinese. The collected samples were immediately cut into small pieces and snap-frozen in liquid nitrogen until further use. All tumor tissue and paired non-cancerous tissue samples were pathologically confirmed.

### RNA extraction and cDNA synthesis

Total RNA from cell lines and liquid-nitrogen-frozen NSCLC tissue samples were extracted using Trizol reagent (Invitrogen). First-strand cDNAs were synthesized using primerscript RT reagent kit (Takara). Briefly, a mix of 2 ug RNA, 2 μl of 5× gDNA eraser buffer and 1 μl of gDNA eraser in a final volume of 10 μl with RNase free DH_2_O, was incubated at 42°C for 2 min to get rid of gDNA, and placed on ice for at least 1 min. Then 4 μL 5× Primerscript buffer, 1 μL Primerscript RT enzyme mix I, 1 μL RT primer mix, and 3 μL RNase free DH_2_O were added, and incubated at 37°C for 15 min followed by 85°C for 5 sec. The cDNA samples were stored at -20°C until use.

### Primer design

Primers were designed using Primer Premier 5.0 and oligo 7 Software. Each primer pair was verified for gene specificity using Nucleotide Basic Local Alignment Search Tool from the GenBank non-redundant nucleotide sequence database (National Centre for Biotechnology Information, 2009, http://www.ncbi.nlm.nih.gov/). The sequence of each primer pair and the expected amplicon size were summarised in Table 1.

**Table 1:** Primer sequences, NCBI gene ID and amplicon size

**Gene**	**NCBI gene ID**		**Sequence(5′—3′)**	**Amplicon size (bp)**
GABR_A3_	2556	Sense	CCGTCTGTTATGCCTTTGTATT	165
		Antisense	TGTTGAAGGTAGTGCTGGTTTT	
GABR_B3_	2562	Sense	GGAGATACCCCCTGGACGAGCA	196
		Antisense	GGATAGGCACCTGTGGCGAAGA	
GABR_E_	2564	Sense	ATGCTTCTCCTAAACTCCGCC	248
		Antisense	CTTAAAACGCTTGCACCACTC	
GABR_P_	2568	Sense	CGACCGTGTTATCAATGACC	120
		Antisense	CCCCAAACACAAAGCTAAAGCA	
GABBR_1_	2550	Sense	CAGATAAATGGATTGGAGGGT	101
		Antisense	GAGAACTGAGACGGAGATAAAGAG	
GABBR_2_	9568	Sense	GAGTCCACGCCATCTTCAAAAAT	108
		Antisense	TCAGGATACACAGGTCGATCAGC	
18S rRNA	100008588	Sense	GCAATTATTCCCCATGAACG	123
		Antisense	GGCCTCACTAAACCATCCAA	

### PCR

PCR was performed to pick out the genes expression in NSCLC cancer cell lines using Premix Taq® version 2.0 kit (Takara). The reaction condition was followed: initial denaturation at 95°C for 1 min; 35 cycles of 30 sec at 95°C; 30 sec at 58°C; and 30 sec at 72°C; followed by a final 3 min extension at 72°C. 3% Agarose gel was used in electrophoresis to separate the reaction products at 80V, 40min. After dyeing with ethidium bromide, the Gel Doc™ EZ Imager (BIO-RAD) was used for imaging.

### RT-qPCR

Real-time quantitative PCR was carried out in tissues cDNA samples using the SYBR® Premix Ex Taq™ reagent kit (Takara) through ABI PRISM®7900 HT Fast Real-Time PCR system (Applied Biosystems). Briefly, 2 μl of each cDNA product was amplified in a mixture containing 12.5 μl of 2 × SYBR® Premix Ex Taq™, 0.5 μl of 10 μmol/L PCR sense primer and the same quantitative antisense primer with dH_2_O in a final volume of 25 μL. The RT-qPCR was performed using the following parameters: initial denaturation at 95°C for 30 sec, 40 cycles of 5 s at 95°C, 30 s at 60°C. The dissolved curve was added to verify the specificity of amplified products. Wells with no template were included for each primer set as a negative control. In each experiment, samples were amplified in duplicate for each of the genes of interest and the reference gene. Only average CT values with a standard deviation <0.5 were accepted.

### Statistic analysis

The expression of each gene of interest was determined in relation to the reference gene 18S rRNA. The difference in the mean CT values of the duplicate samples against the reference gene was calculated to give the ΔCT. The relative quantitation value was then expressed as two times -ΔCT (2^–ΔCT^). Analysis of the 2^–ΔCT^ values data was performed using SPSS 13.0 followed by Paired-Samples *t* Test and Independent-Samples *t* Test where appropriate. Correlation analysis was carried out for GABA concentration and cell growth over time. Survival times between groups were displayed by Kaplan-Meier curves after a log-rank test. *P*<0.05 was considered statistical significance.

## Results

### Effect of GABA on cancer cell proliferation

**Figure 1:**
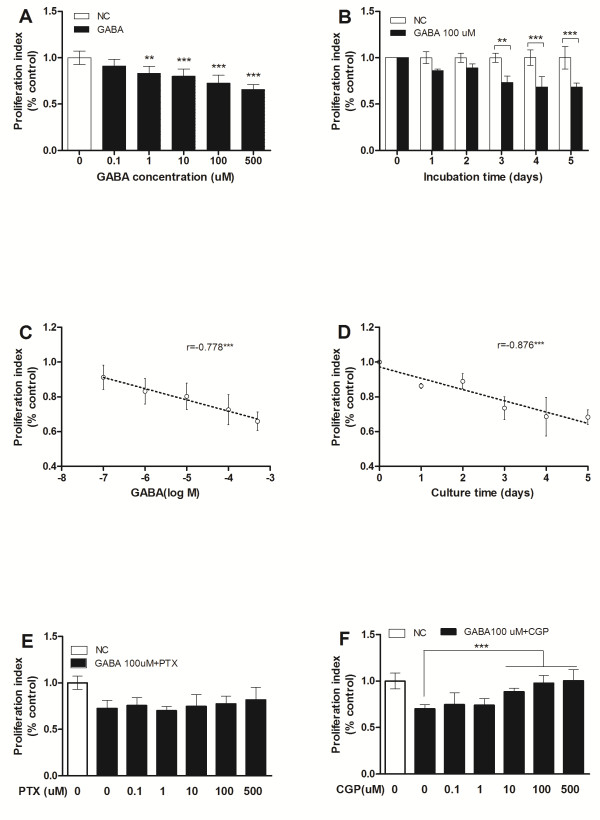
**The effects of GABA and GABA agents on NSCLC cell.** H1299 cell proliferation was suppressed by GABA in a dose-dependent manner measured by MTT assays (Figure 1**A** and **C**). The inhibitory effects of GABA (100 μM/L) on H1299 cell growth were time-dependent (Figure 1**B** and **D**). This inhibitory effects of GABA (100 μM/L) on H1299 cell growth was blocked by CGP co-cultured but not by PTX (Figure 1**E** and **F**). Proliferation index was calculated as the ratio of the absorbance of cells incubated with GABA or GABA agents compare with negative controls (NC) which was defined as the cells cultured only with complete medium. N =3. *P < 0.05, ** *P* < 0.01, *** P < 0.001 vs NC.

To examine the effects of administration of exogenous GABA on cancer cells proliferation in vitro, MTT assays were performed on the H1299 cells at broad range of concentrations of GABA(0.1-500 μmol/L). There was a dose- and time-dependent inhibition of cell proliferation with GABA (Figure 1A, B). The GABA concentration used was negatively correlated with the proliferation index (Figure 1C, r = -0.778, p < 0.001; Figure 1D, r = -0.876, p < 0.001). This inhibitory effects of GABA on cell proliferation was blocked by the use of CGP, a GABA_B_ receptor antagonist, while PTX, a GABA_A_ receptor antagonist, had no effect (Figure 1E, F). Similar results were observed in other NSCLC cell lines, such as A549, H520, H460 (data not show).

### GABA receptor profiles in NSCLC cell lines

**Figure 2:**
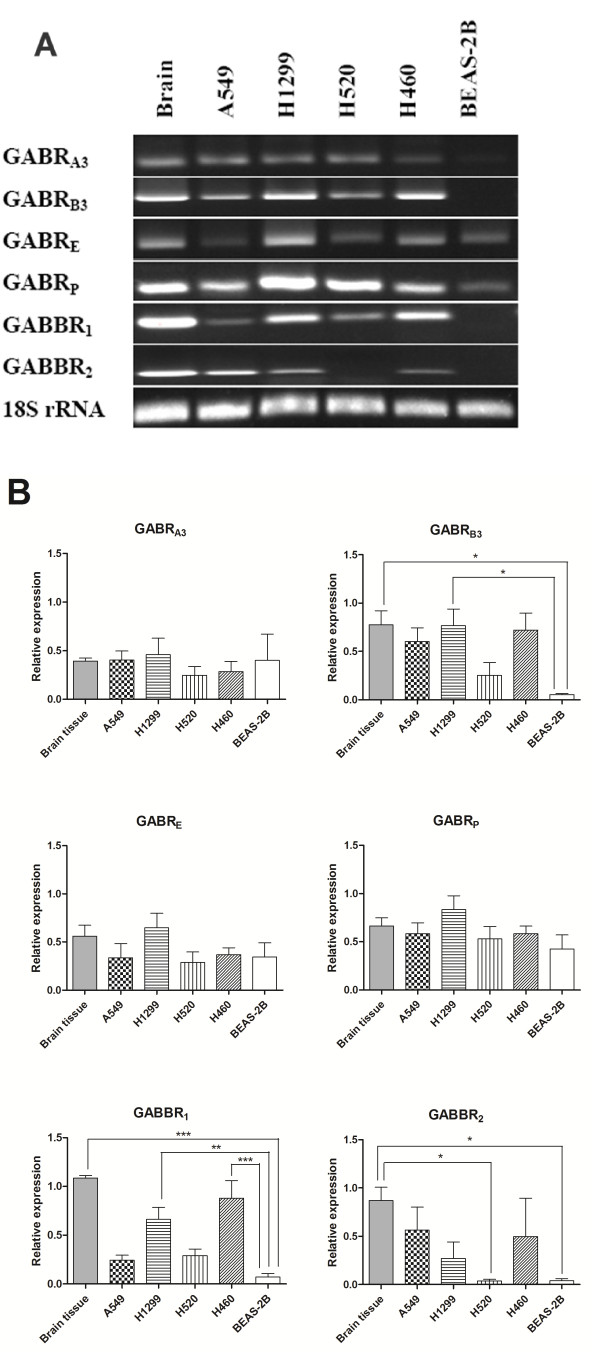
**The mRNA profile of GABA receptors in NSCLC cell lines.** Figure 2**A** show that GABR_A3_, GABR_B3_, GABR_E_, GABR_P_, GABBR_1_ and GABBR_2_ ecpress in NSCLC cell lines. Brain expression acts as a positive control and BEAS-2B as a negative control. Figure 2**B** show three times of the PCR for gene relative expression. * *P* < 0.05, ** *P* < 0.01.

The mRNA profile of GABA receptors was determined in NSCLC cell lines including H1299, A549, H520, H460 and BEAS-2B. The mRNA of human brain (a kind gift from Dr. He Junde, Department of neurosurgery in the same affiliation as ZX) was used as positive control and the normal lung bronchiolial epithelial cells BEAS-2B served as negative control. We found that six GABA receptor subunits were expressed in most NSCLC cell lines including GABA_A_ receptor subunits (GABR_A3_, GABR_B3_, GABR_E_, GABR_P_) and GABA_B_ receptor subunits (GABBR_1_ and GABBR_2_) (Figure 2A and B).

### GABA receptor profiles in NSCLC tissues and paired non-cancerous tissues

**Figure 3:**
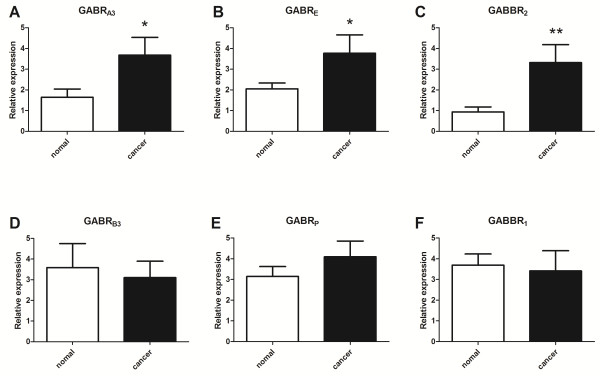
**Gene expression levels in all NSCLC tissues and paired non-cancerous tissues.** Relative expression is presented as 2^-ΔCT^ ± S.E. The expression of 18S rRNA acts as reference gene. There is a significant increase in gene expression for GABR_A3_ (*P = 0.030, Figure 3**A**), GABR_E_ (*P = 0.036, Figure 3**B**) and GABBR_2_ (**P = 0.005, Figure 3**C**) in NSCLC tissues, with no difference in expression for GABR_B3_ (*P* = 0.742, Figure 3**D**), GABR_P_ (*P* = 0.288, Figure 3**E**) and GABBR_1_ (*P* = 0.746, Figure 3**F**). * *P* < 0.05, ** *P* < 0.01.

The gene expression of GABR_A3_, GABR_E_ and GABBR_2_ was significantly higher in primary NSCLC tissues than in the paired non-cancerous tissues (GABR_A3_, *t* = 2.227, *P* = 0.030; GABR_E_, *t* = 2.148, *P* = 0.036; GABBR_2_, *t* = 2.928, *P* = 0.005; Figure 3A, B and C). There was no significant difference observed in the expression of GABR_B3_ (*t* = -0.330, *P* = 0.742), GABR_P_ (*t* = 1.072, *P* = 0.288) or GABBR_1_ (*t* = -0.326, *P* = 0.746, Figure 3D, E and F) in the same tissues examined (Table 2).

**Table 2:** Comparison of GABA receptor gene relative expression between cancer and paired non-cancerous tissue

	**Normal(n = 61)**	**Cancer(n = 61)**	***P *value**
GABR_A3_	1.647 ± 0.394	3.681 ± 0.853	0.030*
GABR_E_	2.054 ± 0.276	3.767 ± 0.883	0.036*
GABBR_2_	0.940 ± 0.236	3.315 ± 0.868	0.005**
GABR_B3_	3.585 ± 1.163	3.103 ± 0.792	0.742
GABR_P_	3.147 ± 0.475	4.095 ± 0.760	0.288
GABBR_1_	3.693 ± 0.538	3.413 ± 0.976	0.746

The data of GABA receptor gene relative expression were expressed as mean ± SE. Normal: Paired non-cancerous tissue. * *P* < 0.05, ** *P* < 0.01.

### Survival analysis in relation to GABA receptor gene profiles

**Figure 4:**
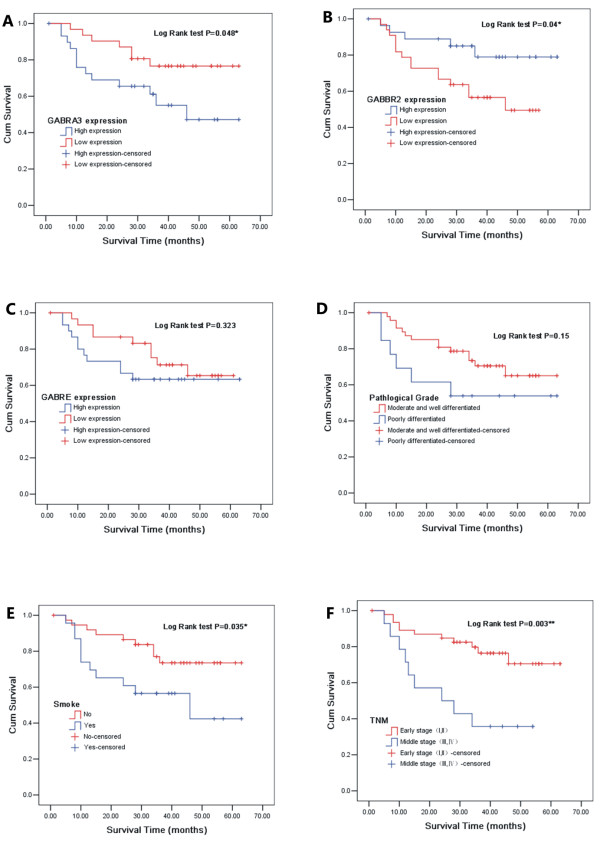
**Kaplan-Meier survival curves of NSCLC patients based on various influential factors.** Figure 4**A**, Overall survival rate in patients with high expression of GABR_A3_ was significantly lower than that in patients with high expression. Figure 4**B**, Overall survival rate in patients with high expression of GABBR_2_ was significantly higher than that in patients with low expression. Figure 4**E**, **F**, the smokers and the patients in advanced stage had a shorter survival time. Figure 4**C**, **D**, In spite of curve separated, GABR_E_ and pathological grade seemed to have no influence on survival time. * *P* < 0.05, ** *P* < 0.01.

Using the median of 2^-ΔCT^ value as cutoff point, the data was divided into high and low expression for analysis. A high expression of GABBR_2_ in the lung tissue was associated with better survival as compared to those where low expression was detected (Figure 4B). However, a higher expression of GABR_A3_ was associated with a lower survival rate (Figure 4A). Smoking and TNM stage were also the important influential factors of decreased survival (Figure 4E, F). Expression of GABR_E_ and pathological grade did not appear to influence on survival time (Figure 4C, D).

### GABA receptors profiles and selected clinical characteristics

The gene expression of GABR_A3_ was significantly higher in the NSCLC tissues in male patients, while the expression of GABBR_2_ was significantly higher than paired controls in the patients of female (Table 3). It appeared that the gene expression of both GABR_A3_ and GABBR_2_ was higher in the patients with early stage of pathology, but not in the advanced stages.

**Table 3:** GABRA3, GABRE and GABBR2 gene expression in NSCLC tissues compare with paired non-cancerous tissues

**Variable**	**N(%)**	***P *value**		
		**GABR**_**A3**_	**GABR**_**E**_	**GABBR**_**2**_
Total	61(100)	0.030*	0.036*	0.005**
Gender				
Male	40(66)	0.040*	NS	NS
Female	21(34)	NS	NS	0.008**
Age				
<60	36(59)	NS	NS	0.046*
>60	25(41)	NS	NS	0.014*
Smoking history				
Yes	23(38)	NS	NS	NS
No	38(62)	NS	NS	0.014*
Histotype				
Adenocarcinoma	43(70)	NS	0.049*	NS
Squamous cell carcinoma	18(30)	NS	NS	0.020*
Pathological Grade				
I, II	48(79)	NS	NS	0.008**
III	13(21)	NS	NS	NS
Pathological Stage				
I, II	47(77)	0.050*	NS	0.008**
III	9(15)	NS	NS	NS
IV	5(8)	NS	NS	NS

Pathological grade I: well differentiation, grade II: moderately differentiation, grade III: poorly differentiation. Pathological stage refers to the 7th Edition of TNM Classification of Malignant Tumors form UICC. *P*-value represents the gene expression of GABA receptor in NSCLC tissues compared with that in paired non-cancerous controls. NS: no significant difference. *P < 0.05, ** *P* < 0.01.

## Discussion

GABA and GABA receptors act as an inhibitory neurotransmitter in the mature CNS, but their functions in non-neuronal cells or tumor cells are not well addressed. Previous investigation has reported that GABA is significantly decreased in NSCLC tissues [26]. The previous observation maybe explained by our current findings demonstrating that GABA exerts inhibitory effects on human NSCLC cell. Our results further demonstrate that GABA_B_ receptors play an important role in mediating the GABA-inhibitory effects on NSCLC cells since the effects were blocked by using the antagonist CGP35348 specific for GABA_B_ receptor. Decreased GABA level might have resulted in impaired inhibitory effects on cancer cell proliferation as seen clinically [26]. A compensatory mechanism may be required by enhancing the expression of GABA_B_ receptors in order to redeem the impaired inhibitory effects.

The etiological factors of lung cancer are still not clear, but the tumor progression is associated with genetic changes and is reflected in phenotypic changes such as altered gene expression profiles. In this study, we found that the six genes of GABA receptors are expressed in most of NSCLC cells and tissues, including GABR_A3_, GABR_B3_, GABR_E_, GABR_P_, GABBR_1_ and GABBR_2_. These subunits might compose functional GABA_A_ and GABA_B_ receptor. These genetic changes suggest that GABA receptors have close relationship with NSCLC progression.

As for GABA_B_ receptors, it is now well accepted that GABA_B_ receptors assemble into heteromers composed of one GABBR_1_ and one GABBR_2_ subunit, which are both required for normal receptor function [6,32]. Some recent reports have suggested that GABA inhibits neoplastic proliferation via GABA_B_ receptor [23,26]. Since GABA_B_ receptor could strongly inhibit base level and isoproterenol-induced cAMP, p-CREB, cyclic adenosine monophosphate response element-luciferase activity and p-extracellular regulated kinase-1 (ERK1)/2 and effectively blocked DNA synthesis and cell migration. The inhibitory cancer cells arrest in G(0)/G(1) phase which is associated with down-regulation of intracellular cAMP level [33]. In our study, GABA inhibited proliferation of NSCLC cells in a dose-dependent and a time-dependent manner. This inhibitory effect could be blocked in the presence of GABA_B_ receptor inhibitor CGP35348. However, co-cultured with GABA_A_ receptor inhibitor picrotoxin, there were no significant proliferative effects on cancer cells versus control. These results imply that GABA inhibition of NSCLC cell proliferation was associated with GABA_B_ receptor which coincides with the report by Schuller and Al-Wadei [23,26]. The high level expression of GABA_B_ receptor gene in NSCLC tissues compared with the adjacent non-tumor lung tissues, implicated that GABA and GABA_B_ receptor pathways could be a critical factor in regulation of NSCLC cells proliferation. This pathway might be a promising molecular target for the development of new therapeutic strategies for antineoplaston.

As for GABA_A_ receptors, it has been demonstrated that GABA_A_ receptors are usually composed of two α subunits, two β subunits, and one γ subunit, and sometime the γ subunit is replaced by other subunits, such as δ, ϵ, π and θ [34]. Different GABA_A_ receptor subunits have been detected in many cancer cell lines and tissues. Li et al. [12] detected overexpression of GABR_Q_ in hepatocellular carcinoma cell line HepG2, and half of the tested hepatocellular carcinoma tissues. Takehara [35] identified the overexpression of GABA receptor pi subunit (GABR_P_) in PDAC cells. In gastric cancer, more than five GABA_A_ receptor subunits were associated with stimulating KATO III cells [28]. The similar results that GABA_A_ receptors are related to cancer cell proliferation were reported in prostate cancer [29], breast carcinoma [36], even in normal human small airway epithelial cells [37]. These findings imply that the inward Cl^—^ ionic current transport and the activated mitogen-activated protein kinase/extracellular signal-regulated kinase (MAPK/Erk) cascade via GABA_A_ receptors positively promotes cell proliferation. Our study revealed that the GABR_A3_ receptor gene was overexpression in NSCLC tissues compare with paired non-cancerous tissues, this was consistent with the study reported by Liu et al. [38]. In addition, beside overexpression of GABR_A3_ and GABR_E_ genes, the other GABA_A_ receptor genes expression in our study including GABR_B3_ and GABR_P_ were also detected in lung cancers, although there was no significant difference between NSCLC tissues and paired non-cancerous tissues. These GABA_A_ receptor subunits may form a functional pentameric chloride channel. We observe that the overexpression GABR_A3_ and GABR_E_ genes were not associated with proliferative effects on cancer cells. This indecipherable phenomenon will lend us to take more investigations of their roles in lung cancers.

Furthermore, our clinical data analysis showed that both GABA_A_ receptors (GABR_A3_) and GABA_B_ receptor (GABBR_2_) genes were significantly expressed in the early pathological stage (stage I and II) of the lung cancer patients, and the expression was gone in stage III and IV. This suggests that high level of gene expression of these GABA receptors may be critical in inhibition of early stage of cancer cells and this regulatory effect got impaired in advanced stage. We speculate that therapeutic intervention approaches that enhance GABA receptors maybe beneficial in late stages of NSCLC patients.

We observe a correlation between the high expression of GABBR_2_ gene and the greater survival rate in patients with NSCLC. The overexpression of GABBR_2_ gene was mostly seen in female patients who had better outcome. This suggests that patients with higher level GABBR_2_ might have better outcome. This observation is consistent with previous in vitro data showing that high level GABBR_2_ gene expression is associated with inhibition of cancer cell proliferation [26,39,40].

In contrast, high level GABR_A3_ gene expression is correlated with cancer cell development [12,35,41], and thus these patients had worse outcome. We indeed showed that the higher gene expression of GABR_A3_ was mostly detected in those male patients who had a worse prognosis.

Our data was based on a relatively small sample size of 61 patients with NSCLC who were followed for 3–5 years. Our data suggests that the gene expression of certain GABA receptor subunits may be useful for prediction of NSCLC prognosis. We believe that a longer term follow-up study with larger sample size would be required to confirm our current findings.

## Conclusion

The present study has identified significant gene profiles of GABA receptors in NSCLC and the gene profiles are correlated with patients’ survival. Exogenous administration of GABA can inhibit NSCLC growth by activation on GABA receptors. Our data suggests that GABA receptors can modulate cancer cell proliferation and their gene profiles may be able to help predict prognosis in patients with NSCLC.

## Competing interests

The authors declare they have no competing interests of this article.

## Authors’ contributions

ZXX participated in all experiments and drafted the manuscript; ZR participated in the RT-qPCR assays; ZYJ, SJF, LJ, SXS, HLY, THL, and LJ carried out collection of the NSCLC tissue, RNA extraction and clinical data analysis; XDK took part in the statistical analysis; HJX participated in the study design; ZHB initiated the study design, participated in data analysis and manuscript writing. All authors read and approved the final manuscript.

## References

[B1] Siegel R Naishadham D Jemal A Cancer statistics, 2012 CA Cancer J Clin 2012 62 10 29 10.3322/caac.20138 22237781

[B2] Jemal A Bray F Center MM Ferlay J Ward E Forman D Global cancer statistics CA Cancer J Clin 2011 61 69 90 10.3322/caac.20107 21296855

[B3] Custodio A Mendez M Provencio M Targeted therapies for advanced non-small-cell lung cancer: current status and future implications Cancer Treat Rev 2012 38 36 53 10.1016/j.ctrv.2011.04.001 21592673

[B4] Sieghart W Structure and pharmacology of gamma-aminobutyric acidA receptor subtypes Pharmacol Rev 1995 47 181 234 7568326

[B5] Kerr DI Ong J GABAB receptors Pharmacol Ther 1995 67 187 246 10.1016/0163-7258(95)00016-A 7494864

[B6] Bettler B Kaupmann K Mosbacher J Gassmann M Molecular structure and physiological functions of GABA(B) receptors Physiol Rev 2004 84 835 867 10.1152/physrev.00036.2003 15269338

[B7] Magnaghi V Ballabio M Cavarretta IT Froestl W Lambert JJ Zucchi I Melcangi RC GABAB receptors in Schwann cells influence proliferation and myelin protein expression Eur J Neurosci 2004 19 2641 2649 10.1111/j.0953-816X.2004.03368.x 15147298

[B8] Ding R Tsunekawa N Obata K Cleft palate by picrotoxin or 3-MP and palatal shelf elevation in GABA-deficient mice Neurotoxicol Teratol 2004 26 587 592 10.1016/j.ntt.2004.04.002 15203181

[B9] Jin N Guo Y Sun P Bell A Chintagari NR Bhaskaran M Rains K Baviskar P Chen Z Weng T Liu L Ionotropic GABA receptor expression in the lung during development Gene Expr Patterns 2008 8 397 403 10.1016/j.gep.2008.04.008 18539546 PMC2581461

[B10] Soltani N Qiu H Aleksic M Glinka Y Zhao F Liu R Li Y Zhang N Chakrabarti R Ng T GABA exerts protective and regenerative effects on islet beta cells and reverses diabetes Proc Natl Acad Sci USA 2011 108 11692 11697 10.1073/pnas.1102715108 21709230 PMC3136292

[B11] Wang FY Watanabe M Zhu RM Maemura K Characteristic expression of gamma-aminobutyric acid and glutamate decarboxylase in rat jejunum and its relation to differentiation of epithelial cells World J Gastroenterol 2004 10 3608 3611 15534915 10.3748/wjg.v10.i24.3608 PMC4612001

[B12] Li YH Liu Y Li YD Liu YH Li F Ju Q Xie PL Li GC GABA stimulates human hepatocellular carcinoma growth through overexpressed GABAA receptor theta subunit World J Gastroenterol 2012 18 2704 2711 10.3748/wjg.v18.i21.2704 22690081 PMC3370009

[B13] Tamayama T Maemura K Kanbara K Hayasaki H Yabumoto Y Yuasa M Watanabe M Expression of GABA(A) and GABA(B) receptors in rat growth plate chondrocytes: activation of the GABA receptors promotes proliferation of mouse chondrogenic ATDC5 cells Mol Cell Biochem 2005 273 117 126 10.1007/s11010-005-8159-6 16013446

[B14] Kanbara K Okamoto K Nomura S Kaneko T Shigemoto R Azuma H Katsuoka Y Watanabe M Cellular localization of GABA and GABAB receptor subunit proteins during spermiogenesis in rat testis J Androl 2005 26 485 493 10.2164/jandrol.04185 15955887

[B15] Andang M Hjerling-Leffler J Moliner A Lundgren TK Castelo-Branco G Nanou E Pozas E Bryja V Halliez S Nishimaru H Histone H2AX-dependent GABA(A) receptor regulation of stem cell proliferation Nature 2008 451 460 464 10.1038/nature06488 18185516

[B16] Lukasiewicz PD Shields CR A diversity of GABA receptors in the retina Semin Cell Dev Biol 1998 9 293 299 10.1006/scdb.1998.0238 9665865

[B17] Watanabe M Maemura K Kanbara K Tamayama T Hayasaki H GABA and GABA receptors in the central nervous system and other organs Int Rev Cytol 2002 213 1 47 11837891 10.1016/s0074-7696(02)13011-7

[B18] Azuma H Inamoto T Sakamoto T Kiyama S Ubai T Shinohara Y Maemura K Tsuji M Segawa N Masuda H Gamma-aminobutyric acid as a promoting factor of cancer metastasis; induction of matrix metalloproteinase production is potentially its underlying mechanism Cancer Res 2003 63 8090 8096 14678958

[B19] Thaker PH Yokoi K Jennings NB Li Y Rebhun RB Rousseau DL Jr Fan D Sood AK Inhibition of experimental colon cancer metastasis by the GABA-receptor agonist nembutal Cancer Biol Ther 2005 4 753 758 10.4161/cbt.4.7.1827 15970706

[B20] Watanabe M Maemura K Oki K Shiraishi N Shibayama Y Katsu K Gamma-aminobutyric acid (GABA) and cell proliferation: focus on cancer cells Histol Histopathol 2006 21 1135 1141 16835836 10.14670/HH-21.1135

[B21] Rotondo A Serio R Mule F Functional evidence for different roles of GABAA and GABAB receptors in modulating mouse gastric tone Neuropharmacology 2010 58 1033 1037 10.1016/j.neuropharm.2010.01.004 20080114

[B22] Al-Wadei HA Ullah MF Al-Wadei M GABA (gamma-aminobutyric acid), a non-protein amino acid counters the beta-adrenergic cascade-activated oncogenic signaling in pancreatic cancer: a review of experimental evidence Mol Nutr Food Res 2011 55 1745 1758 10.1002/mnfr.201100229 21805621

[B23] Al-Wadei HA Al-Wadei MH Schuller HM Cooperative regulation of non-small cell lung carcinoma by nicotinic and beta-adrenergic receptors: a novel target for intervention PLoS One 2012 7 e29915 10.1371/journal.pone.0029915 22253823 PMC3257239

[B24] Schuller HM Al-Wadei HA Neurotransmitter receptors as central regulators of pancreatic cancer Future Oncol 2010 6 221 228 10.2217/fon.09.171 20146581 PMC2832917

[B25] Young SZ Bordey A GABA’s control of stem and cancer cell proliferation in adult neural and peripheral niches Physiology (Bethesda) 2009 24 171 185 10.1152/physiol.00002.2009 19509127 PMC2931807

[B26] Schuller HM Al-Wadei HA Majidi M Gamma-aminobutyric acid, a potential tumor suppressor for small airway-derived lung adenocarcinoma Carcinogenesis 2008 29 1979 1985 10.1093/carcin/bgn041 18310090 PMC2556972

[B27] Roberts SS Mendonca-Torres MC Jensen K Francis GL Vasko V GABA receptor expression in benign and malignant thyroid tumors Pathol Oncol Res 2009 15 645 650 10.1007/s12253-009-9165-x 19381877

[B28] Maemura K Shiraishi N Sakagami K Kawakami K Inoue T Murano M Watanabe M Otsuki Y Proliferative effects of gamma-aminobutyric acid on the gastric cancer cell line are associated with extracellular signal-regulated kinase 1/2 activation J Gastroenterol Hepatol 2009 24 688 696 10.1111/j.1440-1746.2008.05687.x 19032445

[B29] Abdul M McCray SD Hoosein NM Expression of gamma-aminobutyric acid receptor (subtype A) in prostate cancer Acta Oncol 2008 47 1546 1550 10.1080/02841860801961265 18607852

[B30] D’Urso PI D’Urso OF Storelli C Mallardo M Gianfreda CD Montinaro A Cimmino A Pietro C Marsigliante S: miR-155 is up-regulated in primary and secondary glioblastoma and promotes tumour growth by inhibiting GABA receptors Int J Oncol 2012 41 228 234 22470130 10.3892/ijo.2012.1420

[B31] Von Metzler A Nitsch C [Effects of 3-methylcholanthrene and 3-methylcholanthrene plus piracetam on the gamma-amino-butyric acid (GABA) content of several cerebral regions (author’s transl)] J Cancer Res Clin Oncol 1981 101 339 343 10.1007/BF00410120 7309784 PMC12252849

[B32] Pinard A Seddik R Bettler B GABAB receptors: physiological functions and mechanisms of diversity Adv Pharmacol 2010 58 231 255 20655485 10.1016/S1054-3589(10)58010-4

[B33] Wang T Huang W Chen F Baclofen, a GABAB receptor agonist, inhibits human hepatocellular carcinoma cell growth in vitro and in vivo Life Sci 2008 82 536 541 10.1016/j.lfs.2007.12.014 18222491

[B34] Olsen RW Sieghart W GABA A receptors: subtypes provide diversity of function and pharmacology Neuropharmacology 2009 56 141 148 10.1016/j.neuropharm.2008.07.045 18760291 PMC3525320

[B35] Takehara A Hosokawa M Eguchi H Ohigashi H Ishikawa O Nakamura Y Nakagawa H Gamma-aminobutyric acid (GABA) stimulates pancreatic cancer growth through overexpressing GABAA receptor pi subunit Cancer Res 2007 67 9704 9712 10.1158/0008-5472.CAN-07-2099 17942900

[B36] Drell TL Joseph J Lang K Niggemann B Zaenker KS Entschladen F Effects of neurotransmitters on the chemokinesis and chemotaxis of MDA-MB-468 human breast carcinoma cells Breast Cancer Res Trea 2003 80 63 70 10.1023/A:1024491219366 12889599

[B37] Xiang YY Wang S Liu M Hirota JA Li J Ju W Fan Y Kelly MM Ye B Orser B A GABAergic system in airway epithelium is essential for mucus overproduction in asthma Nat Med 2007 13 862 867 10.1038/nm1604 17589520

[B38] Liu Y Guo F Dai M Wang D Tong Y Huang J Hu J Li G Gammaaminobutyric acid A receptor alpha 3 subunit is overexpressed in lung cancer Pathol Oncol Res 2009 15 351 358 10.1007/s12253-008-9128-7 19048400

[B39] Lodewyks C Rodriguez J Yan J Lerner B Lipschitz J Nfon C Rempel JD Uhanova J Minuk GY GABA-B receptor activation inhibits the in vitro migration of malignant hepatocytes Can J Physiol Pharmacol 2011 89 393 400 10.1139/y11-031 21762014

[B40] Schuller HM Al-Wadei HA Majidi M GABA B receptor is a novel drug target for pancreatic cancer Cancer 2008 112 767 778 10.1002/cncr.23231 18098271 PMC3375598

[B41] Liu Y Li YH Guo FJ Wang JJ Sun RL Hu JY Li GC Gamma-aminobutyric acid promotes human hepatocellular carcinoma growth through overexpressed gamma-aminobutyric acid A receptor alpha 3 subunit World J Gastroenterol 2008 14 7175 7182 10.3748/wjg.14.7175 19084931 PMC2776875

